# Kinematic difference and asymmetries during level walking in adolescent patients with different types of mild scoliosis

**DOI:** 10.1186/s12938-024-01211-5

**Published:** 2024-02-19

**Authors:** Run Ji, Xiaona Liu, Yang Liu, Bin Yan, Jiemeng Yang, Wayne Yuk-wai Lee, Ling Wang, Chunjing Tao, Shengzheng Kuai, Yubo Fan

**Affiliations:** 1 School of Biological Science and Medical Engineering, School of Engineering Medicine, Beijing Advanced Innovation Centre for Biomedical Engineering, Beihang University, Beijing, 100191 China; 2 School of Sports Medicine and Rehabilitation, Beijing Sport University, Beijing, 100084 China; 3 Institute of Biomechanics, National Research Center for Rehabilitation Technical Aids, Beijing, 100176 China; 4 Department of Spine Surgery, Shenzhen Second People’s Hospital, Shenzhen, 518039 China; 5 Department of Spine Surgery, First Affiliated Hospital of Shenzhen University, Shenzhen, 518035 China; 6 Shenzhen University School of Medicine, Shenzhen, 518060 China; 7 Shenzhen Youth Spine Health Center, Shenzhen, 518035 China; 8 Department of Orthopedics and Traumatology, The Chinese University of Hong Kong, Hong Kong, SAR China; 9 Tianjin Key Laboratory for Advanced Mechatronic System Design and Intelligent Control, School of Mechanical Engineering, Tianjin University of Technology, Tianjin, 300384 China; 10 National Demonstration Center for Experimental Mechanical and Electrical Engineering Education, Tianjin University of Technology, Tianjin, China

**Keywords:** Adolescent idiopathic scoliosis, Gait symmetry, Curve number, Kinematics

## Abstract

**Background:**

Adolescent idiopathic scoliosis (AIS), three-dimensional spine deformation, affects body motion. Previous research had indicated pathological gait patterns of AIS. However, the impact of the curve number on the walking mechanism has not been established. Therefore, this study aimed to compare the gait symmetry and kinematics in AIS patients with different curve numbers to healthy control.

**Results:**

In the spinal region, double curves AIS patients demonstrated a smaller sagittal symmetry angle (SA) and larger sagittal convex ROM of the trunk and lower spine than the control group. In the lower extremities, the single curve patients showed a significantly reduced SA of the knee joint in the frontal plane, while the double curves patients showed a significantly reduced SA of the hip in the transverse plane.

**Conclusion:**

The curve number indeed affects gait symmetry and kinematics in AIS patients. The double curves patients seemed to adopt a more "careful walking" strategy to compensate for the effect of spinal deformation on sensory integration deficits. This compensation mainly occurred in the sagittal plane. Compared to double curves patients, single curve patients unitized a similar walking strategy with healthy subjects.

## Background

Adolescent idiopathic scoliosis (AIS) is a common three-dimensional deformation of the spine, which has been reported to be prevalent in 1–3% [1–3] of the population aged 10–16 years. To date, the etiology of AIS remains unclear. However, there are several hypotheses regarding the genetic, biomechanical, neurological, and muscular factors. From the view of biomechanics, previous studies have found that AIS could alter postural orientation and induce abnormal gait patterns [4–9]. As a result, the investigation of the walking pattern between healthy subjects and AIS patients may help better understand AIS's etiology.

Since the AIS is a kind of spinal deformity, the trunk motion could be affected directly. It was found that AIS patients had a smaller spinal range of motion (ROM) [4]. Recent studies have even exhibited the variation of trunk motion according to the severity and type of scoliosis [10–13]*.* In specific, trunk motion parameters were correlated with Cobb angle at 0.82. In Nishid’s study, the AIS patients with a single thoracic curve showed asymmetrical trunk movement in the transverse plane, and patients with a single lumbar curve showed asymmetrical trunk movement in the coronal plane. However, in Pesenti’s study, the AIS patients with a single thoracic or lumbar curve presented no difference in the transverse plane. Apart from the trunk motion, the postural adjustment of AIS patients during level walking also involved the pelvis, upper extremities, and lower extremities. Wu et al. [14] found that the kinematics of the trunk, pelvis, and lower extremities differed between concave and convex sides at different gait events in severe thoracic AIS. Also, the AIS patients demonstrated reduced ROM in the upper body and lower extremities [9, 15]. However, the previously observed differences were inconsistent, and most studies were limited to single curve AIS or mixed up all curve types and Cobb angles. Moreover, the trunk was regarded as a rigid body and the relative motion between spinal vertebrae was omitted.

This study aimed to investigate the symmetry and kinematics of the multi-spine and lower extremities among healthy subjects, single curve AIS patients, and double curves AIS patients during a stance phase of gait. Our first hypothesis was that AIS patients might demonstrate more kinematic asymmetry than healthy subjects. The second hypothesis was that the kinematic characteristics of multi-spine and lower extremities in three planes might vary with the type of AIS.

## Methods

### Subjects

**Fig. 1:**
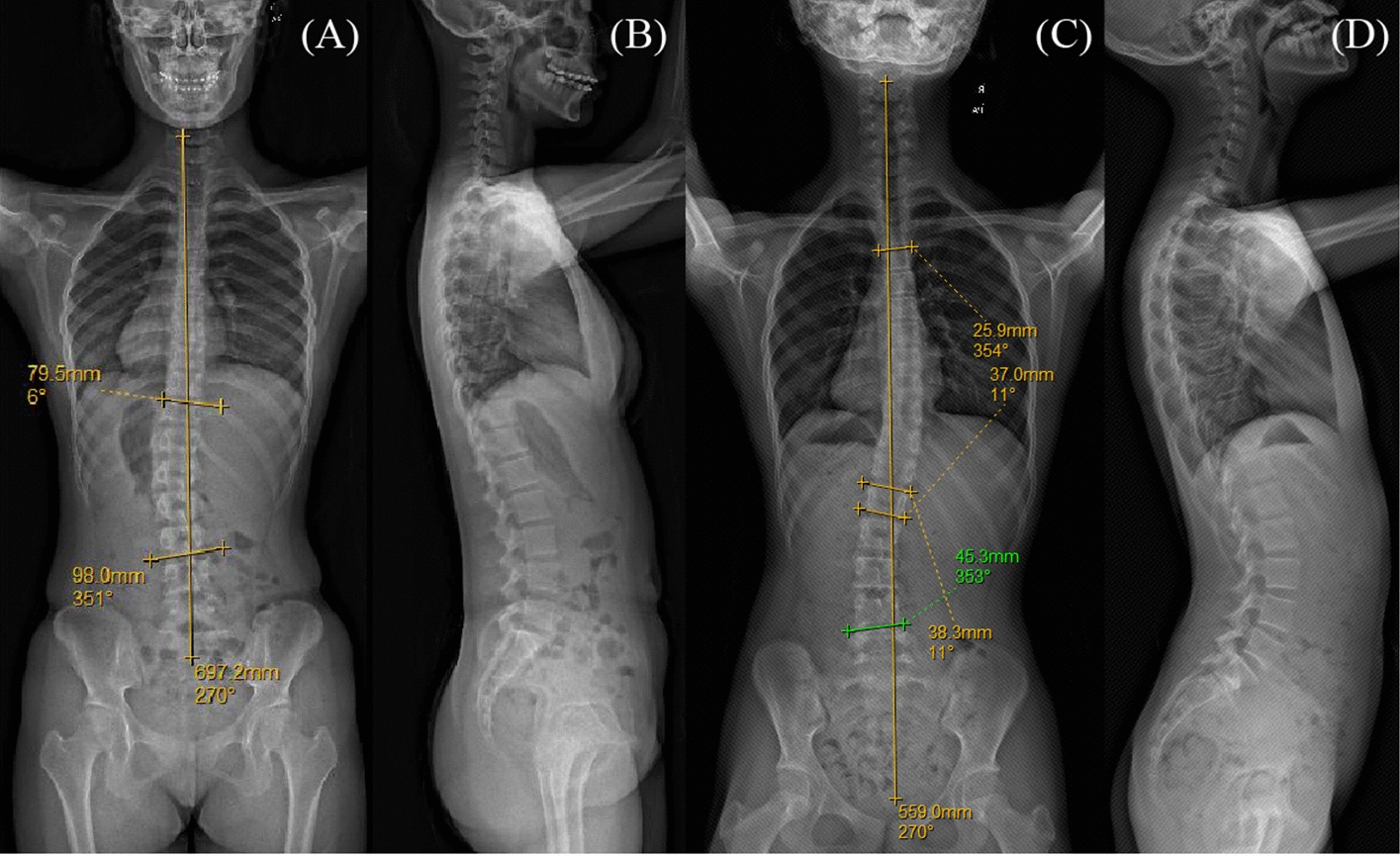
Frontal and lateral radiographic images of one PUMCI patient (**A**, **B**), frontal and lateral radiographic images of one PUMCII patient (**C**, **D**)

From January 2022 to September 2022, 36 mild AIS patients and 18 control teenagers were recruited for this study. The criteria for the inclusion were as follows: (1) diagnosed as AIS through radiological examination, (2) Cobb’s angle ranges from 10 to 25 degrees, and (3) aged 8–16 years. The scoliosis patient was further divided into PUMCI (single curve) and PUMCII (double curves) according to the PUMCI classification [16] (Fig. 1), and those patients that did not belong to the two groups were excluded from the study. Specially, the PUMCII patients with identical primary and secondary Cobb angles were also excluded. Moreover, any subject who has metabolic or oncological or chronic respiratory system diseases, central nervous system disorders, injuries, and fractures within the previous 6 months was also excluded. The control group included teenagers aged 8–16 years without systemic diseases and with an angle of ATR less than 5°. The general information of these participants is shown in Table 1. The study was approved by the Ethics Committee of Shenzhen Second People’s Hospital and conducted according to the Declaration of Helsinki. All participants and their parents signed an informed consent form before the experiment.

**Table 1:** Demographics of participants

Group	Age (Year)	Height (cm)	Weight (kg)	Cobb Angle (°)
Control	11.27 ± 2.03	150.16 ± 13.97	40.44 ± 11.57	–
PUMCI	12.66 ± 2.63	155.16 ± 15.74	42.55 ± 11.28	15.66 ± 2.54
PUMCII	13.61 ± 1.37	159.94 ± 6.84	42.50 ± 7.84	16.11 ± 2.21

### Procedure

**Fig. 2:**
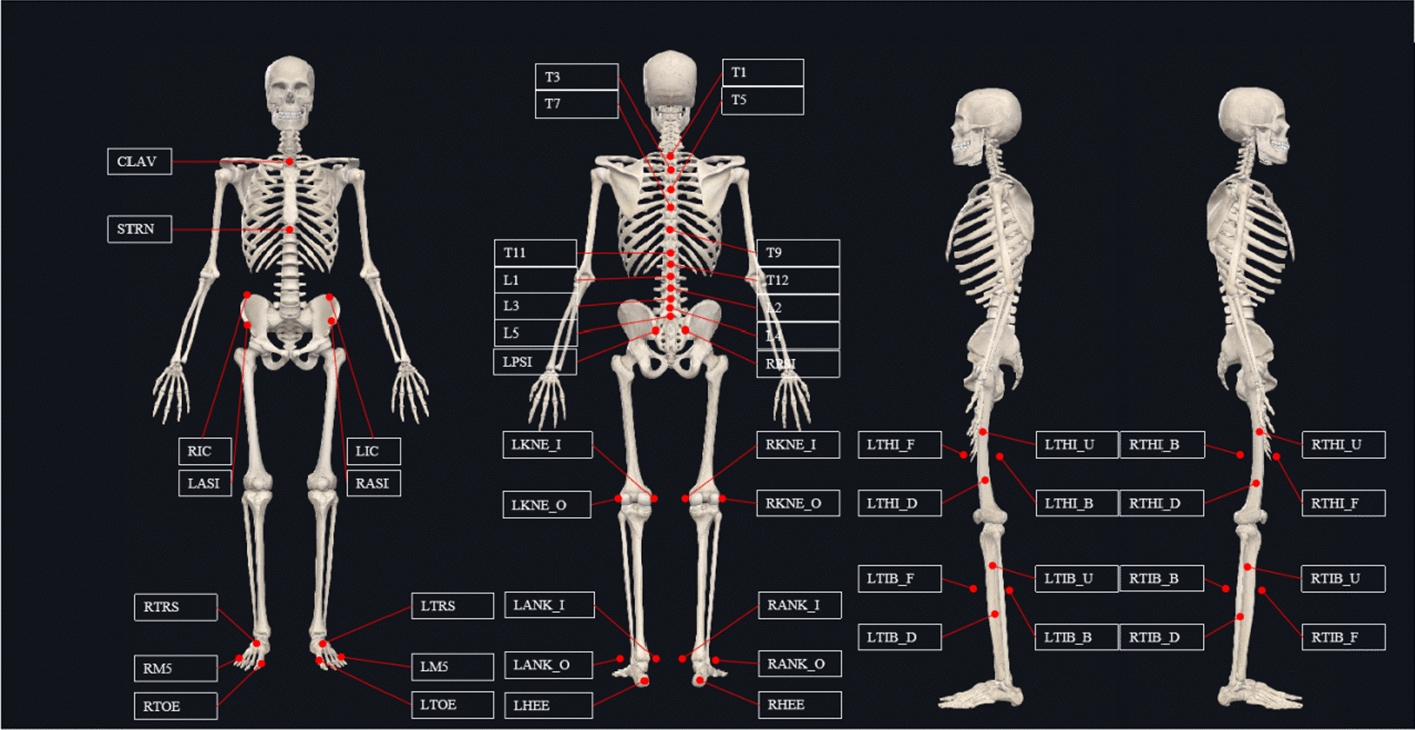
The schematic of the marker set definition in this study

Before the experiment, 56 reflective markers were placed on the landmarks according to the definition of Fig. 2. In the spinal region, the markers were placed on the jugular notch where the clavicles meet the sternum (clavicle, CLAV), the xiphoid process of the sternum (sternum, STRN), the spinous process of the first, third, fifth, seventh, ninth, eleventh and twelfth thoracic vertebrae (T1, T3, T5, T7, T9, T11, T12), of the first, second, third, fourth, fifth lumbar vertebrae (L1, L2, L3, L4, L5). In the pelvic region, the markers were placed on the left and right anterior superior iliac (LASI, RASI), left and right posterior superior iliac (LPSI, RPSI), and left and right iliac crest (LIC, RIC). In the thigh region, two markers were placed on the medial and lateral condyle of the femur (KNE_I, KNE_O), and non-collinear four makers were placed on the surface of the thigh (THI_U, THI_D, THI_F, THI_B). In the shank region, two markers were placed in the medial and lateral malleolus, and non-collinear four makers were placed on the surface of the shank (TIB_U, TIB_D, TIB_F, TIB_B). In the foot region, the markers were placed on the first and third metatarsal head (Toe, M5), calcaneus (HEE), and superior surface of the cuneiform bone (TRS).

After marker placement, participants assumed an anatomical pose for 1 min to get a standing reference trail, then they were asked to walk through a 10 m walkway at their self-selected speed for several minutes to adapt to this experiment. Subsequently, participants walked naturally for data collection. During level walking, a ten-camera three-dimensional (3D) motion capture system (MotionAnalysis Corp., USA) and two force platforms (AMTI Inc., Watertown, MA, USA) embedded in the walkway were used to collect three-dimensional markers’ trajectories and ground reaction force (GRF) with a sampling frequency of 100 Hz and 1000 Hz, respectively.

### Musculoskeletal model and simulation

A generic MoCap_FullBody model in the AnyBody modeling system (version 6.0.6, Aalborg, Denmark) was selected, and the upper extremities were removed for this study. Therefore, the musculoskeletal model contained the spine, pelvis, and lower extremities. The spinal model consisted of five lumbar vertebrae and one lumped thoracic segment. The pelvis was one lumped segment. The lower extremities were composed of the thigh, shank, and foot. These segments were linked by joints with 35 degrees of freedom (DOFs). In specific, two spherical hip joints (with 3 DOFs) that linked pelvis and thigh, two spherical knee joints (with 3 DOFs) that linked thigh and shank, two revolute ankle joints (with 1 DOF) that linked the shank and foot, six spherical interverbal joints (with 3 DOFs) that linked the spinal segments, and one spherical sacroiliac joint (with 3DOFs) that linked the spine and pelvis. The positions of these joints were pre-defined in the MoCap_FullBody model, which were based on the work by Pearcy and Bogduk [17]. A detailed overview and validation of the model were given in the literature [18–20].

For each participant, the standing reference trial was utilized to identify the parameters of segment lengths and the (virtual) marker positions of the musculoskeletal model. Then, inverse kinematics was applied to minimize the errors between captured markers and virtual markers, driving the musculoskeletal model to simulate the participant walking.

In general, a minimum of three markers was essential to determine the segmental motion. For pelvis and lower extremities, the markers attached to these segments were enough to drive the motion of these segments. However, there were insufficient markers to determine the vertebral bodies. Consequently, the spine model followed a spine rhythm that distributed the trunk motion over the vertebral bodies in AnyBody modeling system. In this study, the spine rhythm was removed and each vertebral body was independently driven by captured markers referred to the previous studies [21]. In specific, the thorax was driven by CLAV, STRN, T1, T3, T5, T7, T9, T11 and T12. The first lumbar vertebra was driven by T12, L1, and L2, while other spinal vertebrae were driven using a similar method.

### Data analysis

After walking simulation, the joint angles of lower extremities and multi-spine were the output of the musculoskeletal model. The convex angular ROM of the multi-spine and lower extremities were computed for the stance phase of gait, defined from convex heel strike to convex toe-off. The concave ROM was calculated similarly. The term "convex" refers to the side of the major scoliosis curve in both PUMCI and PUMC II, while "concave" refers to the opposite side. The degree of symmetry between the convex and concave ROM was assessed using the symmetry angle (SA) [22], with a score of 0% indicating perfect symmetry and 100% indicating perfect asymmetry.

The results of the Shapiro–Wilk tests indicated that the majority of demographics, convex ROM and SA did not follow a normal distribution. As a result, Kruskal–Wallis’s test was applied to compare the demographics, SA, and convex ROM across the three groups. All statistical analyses were conducted using a custom MATLAB program (The Math Works, Natick, MA, USA). The level of statistical significance was set at *p* < 0.05.

## Result

**Fig. 3:**
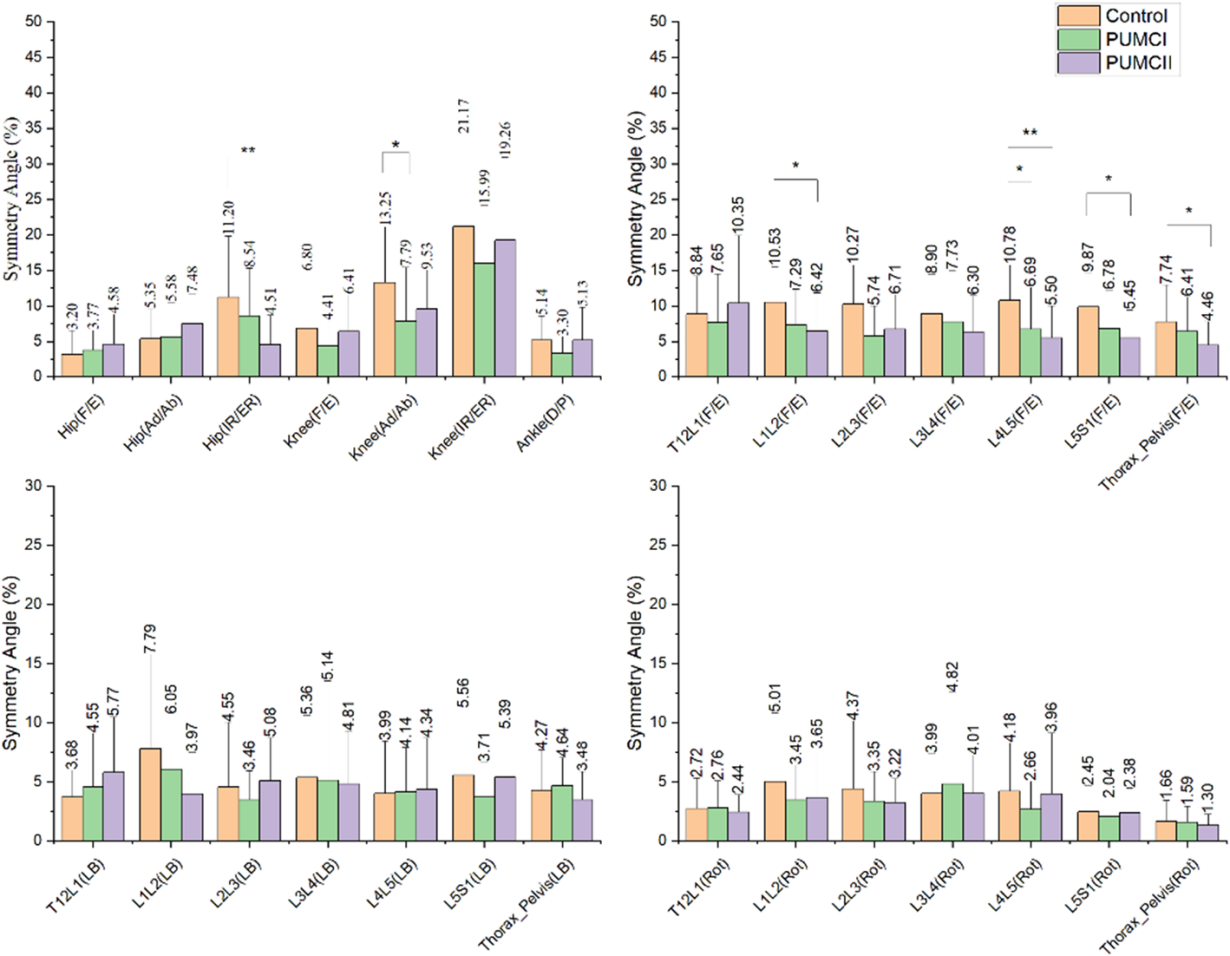
SA scores for the joint angle of lower extremities and multi-spine. ** indicates *p* < 0.001; * indicates *p* < 0.05 (F/E: flexion/bending; Ad/Ab: adduction/abduction; IR/ER: internal rotation/external rotation; D/P: dorsiflexion/plantarflexion; LB: lateral bending; Rot: rotation; Thorax_Pelvis: thorax with respect to pelvis; Thorax_Pelvis: thorax with respect to Pelvis; T12L1: thorax with respect to first lumbar vertebra; L1L2: first lumbar vertebra with respect to second lumbar vertebra; L2L3: second lumbar vertebra with respect to third lumbar vertebra; L3L4: third lumbar vertebra with respect to forth lumbar vertebra; L4L5: forth lumbar vertebra with respect to fifth lumbar vertebra; L5S1: fifth lumbar vertebra with respect to sacrum)

**Fig. 4:**
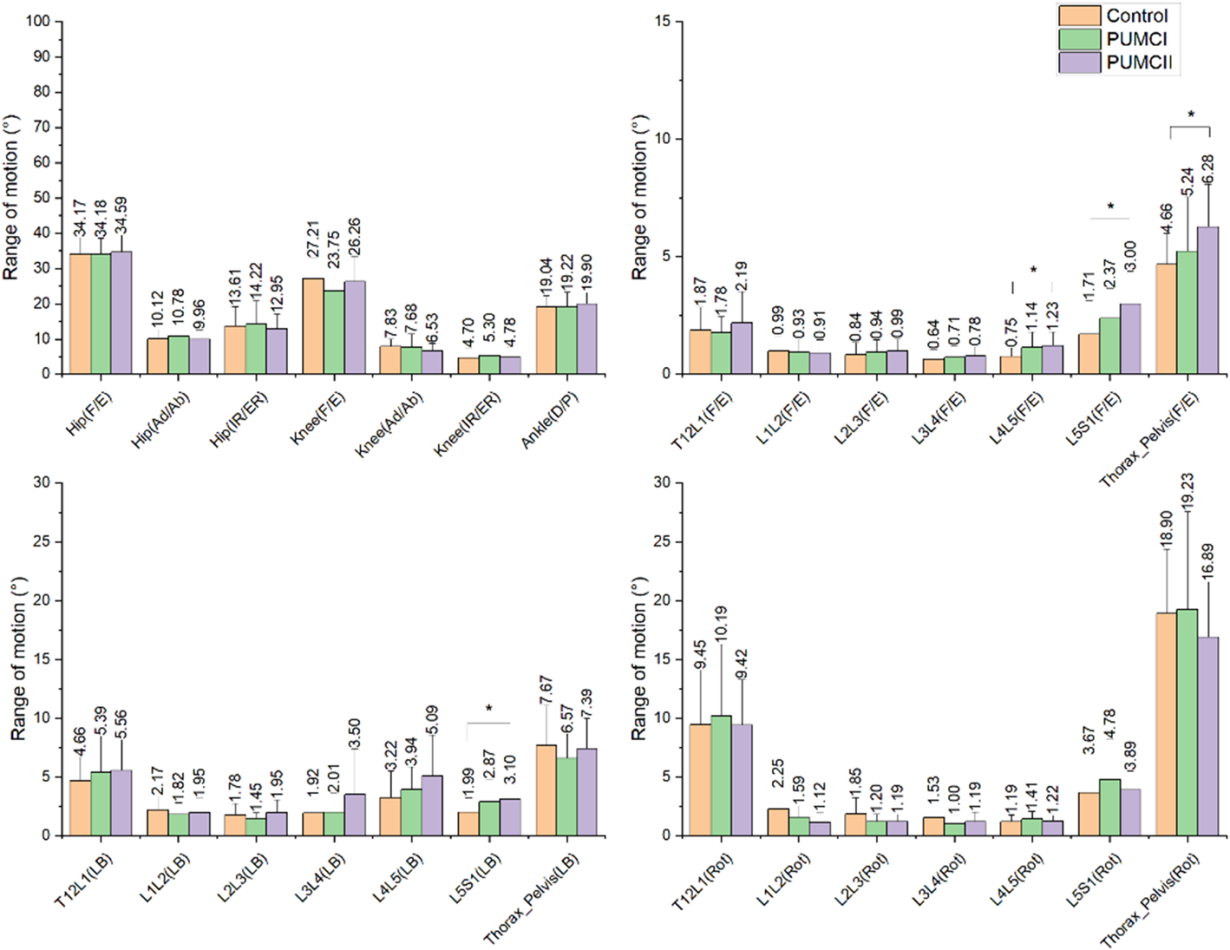
ROM of the joint angle of lower extremities and multi-spine in the convex side. *p < 0.05

Table 1 shows that there is no significant difference in age, height, weight, and cobb angle among the three groups. Figures 3 and 4 demonstrate the symmetry and convex kinematics of the lower extremities and multi-spine in the three planes during a stance phase of gait.

In the sagittal plane, no significant differences in symmetry and convex ROM of lower extremities were observed among the three groups. However, a significant reduction in SA of L1L2 between control and PUMCII (*p* < 0.05), of L4L5 between control and PUMCI (*p* < 0.05), between control and PUMCII (*p* < 0.01), of L5S1 between control and PUMCII (*p* < 0.05), of Thorax_Pelvis between control and PUMCII (*p* < 0.05). In addition, the convex ROM of L4L5, L5S1, and Thorax_Pelvis increased significantly in PUMC II patients.

In the frontal plane, the knee SA decreased significantly and convex L5S1 ROM on the convex side increased significantly.

In the transverse plane, a reduced SA of the hip was found between the control and PUMCII (*p* < 0.01).

## Discussion

The main objective of this study was to compare the kinematic symmetry and convex kinematics of multi-spine and lower extremities between healthy adolescents and two types of mild AIS patients. Contrary to our first hypothesis, the AIS patients exhibited a smaller SA of ROM, suggesting more symmetry in spinal and lower extremities motion during level walking. In addition, no significant differences in convex ROM were found between the control and PUMCI, and a significant increase in convex ROM was observed between the control and PUMCII, which supports the second hypothesis.

In the sagittal plane, no significant difference in the SA and convex ROM was found in the lower extremities during level walking among the three groups, indicating mild AIS did not affect the sagittal movement of the lower extremities. The finding of no symmetry difference among groups was in agreement with previous studies [5, 9, 23]. Therefore, the AIS patients seemed to utilize the same sagittal symmetric walking strategy as the control group in previous studies and our studies. As for the spine, the SA of Thorax_Pelvis showed a significant reduction in PUMC II. Further, our multi-spine model clarified that the reduction was found in L1L2, L4L5, and L5S1 in detail. Although no significant reduction of SA was observed in Thorax_Pelvis between PUMCI and control, the PUMC I demonstrated a significant SA reduction of L4L5, which provided new insight into the symmetry between groups. The convex ROM of Thorax–Pelvis was found to increase in PUMCII. In specific, the increase occurred at the L4L5 and L5S1. However. No significant difference was found between PUMC I and control. These findings were in contrast with previous studies that no difference between control and AIS patients was observed in sagittal trunk motion [5, 9], which might be due to the simple trunk model and mix-up classification of AIS. Similarly, this study mixed up the different apex locations of PUMC I. However, the location of the apex for single curve AIS did not affect the sagittal trunk motion [12]. Therefore, it could be inferred that the two types of AIS patients adopted different walking strategies in the sagittal plane, and the strategy of double curves AIS patients seems to be more careful because of the smaller SA.

In the frontal plane, the PUMCI demonstrated a significant reduction of SA in the knee joint compared to the control, which is inconsistent with previous findings [5, 9]. Mahaudens et al. [5] found that the frontal difference of the lower extremities occurred at the hip joint due to the prolonged electrical activity duration of the related muscles. In contrast, no difference was found in Yang’s study [9]. The reduced SA of the knee joints in the single curve patients may be considered abnormal muscle activities of the semitendinosus and lateral heads of Gastrocnemius [24] and a compensation for the alteration of the center of body mass (COM). In the spinal region, no significant difference in SA was found among the three groups, which was in agreement with Yang’s research [9]. Moreover, the convex ROM of Thorax_Pelvis also did not show the difference among the three groups, in contrast to findings that scoliosis patients had significantly reduced Thorax_Pelvis in the frontal plane [5, 23]. The patients in this study were mild, and the effect of AIS on the trunk oblique might not be enough to be detected. However, the multi-spine model of this study revealed that L4L5 and L5S1 of PUMCII tended to have a larger convex ROM and the significant difference was observed in L5S1, which was similar to sagittal findings that double curves patients showed larger ROM of the lower spine. Therefore, a multi-spine model was essential when assessing the kinematic characteristics in mild AIS patients and could provide new insights on the AIS patients’ kinematics.

In the transverse plane, only a significant reduction of hip SA was found between PUMC II and the control, which agreed with the previous study [5]. However, previous research showed that scoliosis patients had significantly reduced trunk ROM and asymmetric trunk rotation in the transversal plane compared to normal subjects [5, 9, 23]. Lots of previous studies had presented the “torsional offset” in AIS patients and found the torsional motion could be affected by the position of the major curve [5, 11, 25]. This study showed the tendency to be more symmetry in the transverse plane. However, no significant difference was detected since the major curve's position was not considered in this study.

There are several limitations to this study. First, only two types of PUMC were included and the subgroup of PUMC I and PUMCII may affect the result of the kinematic findings. Second, we did not analyze muscle activities and kinetic data such as joint force applied at different spine levels. This is important to understand the AIS mechanism of postural control. Third, the upper extremities were not included in this study since the upper extremities play an essential role in body balance and walking strategy. Fourth, the motion between thoracic vertebrae was omitted in the musculoskeletal model, which might provide new insights into the spinal motion of AIS patients.

## Conclusions

The AIS patients adopted a more symmetry gait strategy to compensate for the larger ROM of the multi-spine and lower extremities. The primary compensation occurred in the sagittal plane and lower spine in specific. Compared with PUMC II, the kinematics of PUMCI was closer to the control, suggesting the single curve AIS was more stable than the double curves AIS. The finding of this study may contribute to the understanding of the kinematic characteristics of lower extremities and spinal motion during the progression of AIS and also had the potential to help the development of rehabilitation and treatment plans.

## Data Availability

Not applicable.
